# Spatial ability and 3D model colour-coding affect anatomy performance: a cross-sectional and randomized trial

**DOI:** 10.1038/s41598-023-35046-2

**Published:** 2023-05-15

**Authors:** Ming Yi Koh, Gerald Jit Shen Tan, Sreenivasulu Reddy Mogali

**Affiliations:** 1 Lee Kong Chian School of Medicine, Nanyang Technological University Singapore, 11, Mandalay Road, Singapore, 308232 Singapore; 2 Diagnostic Radiology, Tan Tock Seng Hospital, Singapore, Singapore

**Keywords:** Anatomy, Medical research

## Abstract

Photorealistic 3D models (PR3DM) have great potential to supplement anatomy education; however, there is evidence that realism can increase cognitive load and negatively impact anatomy learning, particularly in students with decreased spatial ability. These differing viewpoints have resulted in difficulty in incorporating PR3DM when designing anatomy courses. To determine the effects of spatial ability on anatomy learning and reported intrinsic cognitive load using a drawing assessment, and of PR3DM versus an Artistic colour-coded 3D model (A3DM) on extraneous cognitive load and learning performance. First-year medical students participated in a cross-sectional (Study 1) and a double-blind randomised control trial (Study 2). Pre-tests analysed participants' knowledge of anatomy of the heart (Study 1, *N* = 50) and liver (Study 2, *N* = 46). In Study 1, subjects were first divided equally using a mental rotations test (MRT) into low and high spatial ability groups. Participants memorised a 2D-labeled heart valve diagram and sketched it rotated 180°, before self-reporting their intrinsic cognitive load (ICL). For Study 2, participants studied a liver PR3DM or its corresponding A3DM with texture-homogenisation, followed by a liver anatomy post-test, and reported extraneous cognitive load (ECL). All participants reported no prior anatomy experience. Participants with low spatial ability (*N* = 25) had significantly lower heart drawing scores (*p* = 0.001) than those with high spatial ability (*N* = 25), despite no significant differences in reported ICL (*p* = 0.110). Males had significantly higher MRT scores than females (*p* = 0.011). Participants who studied the liver A3DM (*N* = 22) had significantly higher post-test scores than those who studied the liver PR3DM (*N* = 24) (*p* = 0.042), despite no significant differences in reported ECL (*p* = 0.720). This investigation demonstrated that increased spatial ability and colour-coding of 3D models are associated with improved anatomy performance without significant increase in cognitive load. The findings are important and provide useful insight into the influence of spatial ability and photorealistic and artistic 3D models on anatomy education, and their applicability to instructional and assessment design in anatomy.

## Introduction

Anatomy has traditionally been delivered and conducted through cadaveric dissections^1^. However, there is an increasing demand for alternatives teaching methods due to financial, emotional, safety, and practical challenges associated with the use of cadavers^2–4^. While technology has given rise to new methods, these must be evaluated for their effectiveness as realistic supplements to anatomy teaching and learning.

One such method is using three-dimensional (3D) models, which includes both digital and physical models, which can be manipulated spatially to enhance visualisation of structures and their relationships^5^. Their increased accessibility and improved user-interaction^6^ has allowed for increased performance in tasks involving spatial ability (SA) and structural recognition^7–9^. However, cost and decreased authenticity remain key disadvantages in terms of applicability and reproducibility^6^.

An example of a 3D anatomy model is a photorealistic one produced via photogrammetry. Photorealistic 3D models (PR3DMs) are produced by taking a series of pictures from different angles to gather spatial information about an object^10,11^. Hence, photorealistic 3D models can be defined as digital replicas of real-world objects that look the same as their physical counterparts from a human's point of view^10^. Photogrammetry’s advantages over other 3D models are that they can be rotated, altered digitally, and viewed from different perspectives^6^.

There is conflicting evidence about the effects of PR3DMs in anatomy^12,13^. Perception of realistic objects has been explained through geon theory, where primitive 3D shapes are geons, perceptual units used in segmenting and processing visualisations^14,15^. Thus, the perception of 3D objects may be hindered by complex, realistic surfaces that have more geons. Several studies have reported higher cognitive load and worse learning performances in realism due to higher perceptual complexity^12,16^, as extraneous details impair both learning and transfer of information^17^. However, there was a report of the paradoxical effect of realism, explained through the disfluency theory that realism better captures learners’ attention and aids information retrieval, improving learning performance^13,18,19^.

The inherent difficulty of a subject contributes to intrinsic cognitive load (ICL) while the nature of content presentation that is designed counterproductively imposes extraneous cognitive load (ECL)^20^. The overall cognitive load of a visualisation is defined as the summation of ICL and ECL^21^. Visual signalling can minimise ECL of photorealistic visualisations^14,22–24^, assisting students by modelling more effective search strategies and directing learners to crucial elements^25–27^. These cues may be additive (arrows, pointers etc.) or non-additive (background blurring, colour-coding etc.)^28^. Colour-coding is an established signalling strategy that has been repeatedly shown to positively-affect learning, including in information retention, transfer and matching^29–31^, while minimising ECL^32,33^. Lokka and Çöltekin had shown the benefits of combining realism with abstraction on recall accuracy and learning performance^34^, and a similar method was suggested to be applied to anatomy education^14^. However, there is limited information regarding effects of colour coding PR3DMs on anatomy learning performance and ECL. To our knowledge, there is only one study reporting the effect of adding the signalling effect of colour-coding to PR3DMs on learning performance, but it was not based on a structure that exists in human anatomy^14^.

Spatial ability (SA), the ability to retain, generate, retrieve and transform well-structured visualisations^35^, and its effects on anatomy learning have been widely studied. Several studies have shown positive correlations between SA and anatomy knowledge, hypothesised to be because anatomy requires firm comprehension of 3D relationships between anatomical structures, especially when viewed from different perspectives^36–38^. Spatial ability is also important for one to produce mental 3D images based on the assimilation and synthesis of two-dimensional imagery^39^. However, very few studies involved drawing as a measure of post-intervention knowledge, a method that provides comprehensive insight into learners’ mental 3D-representation of structures^40^, of which only one was about human anatomy^41^ and another related to cross-sectional anatomy of the dog^42^. Thus, there remains lack of guidance and consensus for the design of anatomy courses based on SA.

### Objective of the current study

This study aimed to investigate how spatial ability affects anatomy learning using drawing assessment, and the effects of colour coded PR3DMs on extraneous cognitive load and learning performance. We hypothesised that (1) learners with high spatial ability would have better anatomy performance in drawing assessment, (2) colour coded PR3DM (i.e., A3DM) of the liver will decrease ECL, and (3) learning with A3DM models would results in better anatomy performance. Gaining a deeper understanding about these will hopefully aid in the design of tools and curriculum for anatomy teaching and learning, including possible customisation based on individual needs and abilities.

## Methods

### Study design

**Figure 1:**
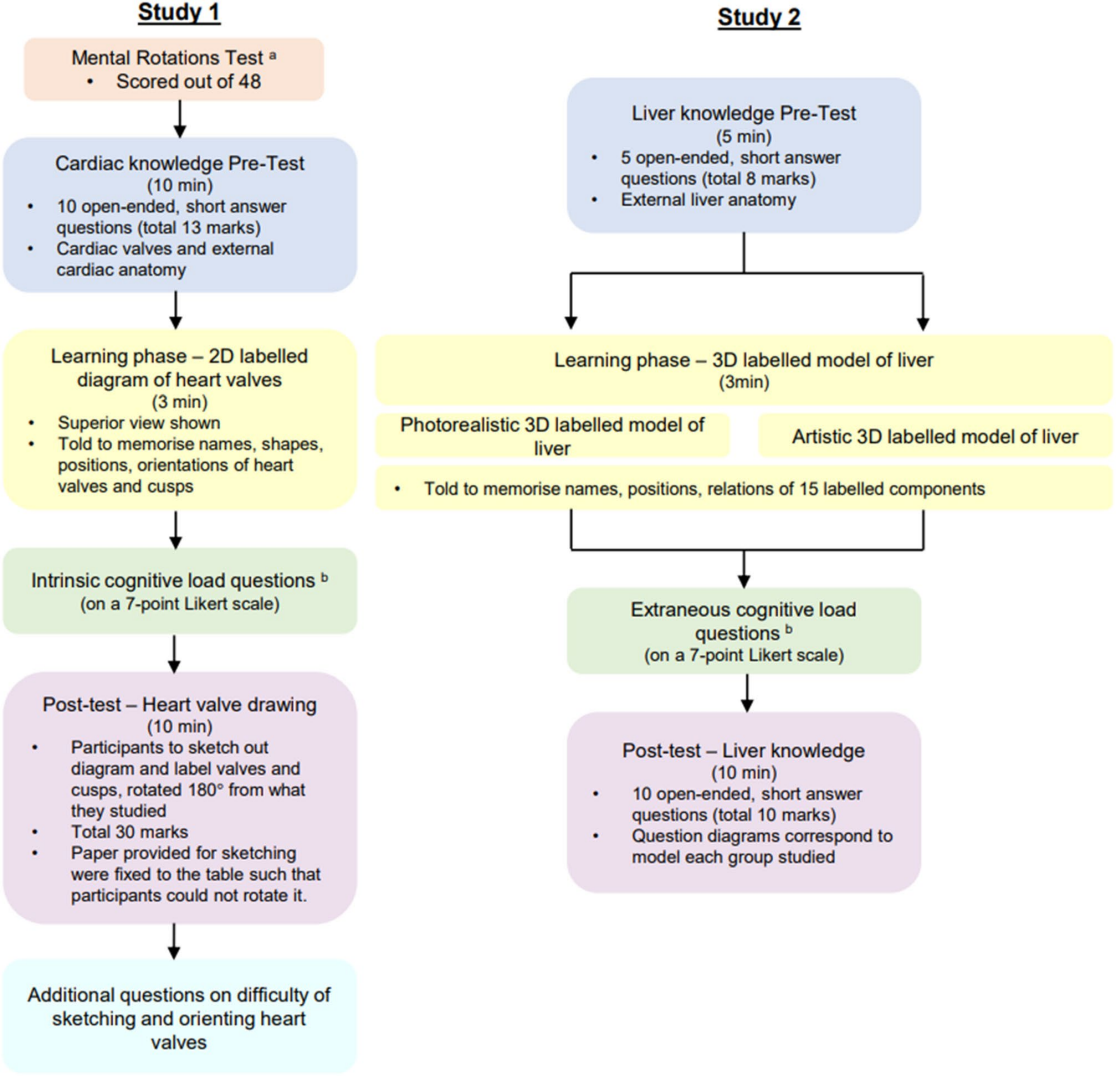
Flowchart depicting the conduct of studies 1 and 2. ^a^Redrawn Vandenberg & Kuse (1978) mental rotations test developed by Peters et al. (1995)^43^. ^b^Cognitive load survey instrument by Klepsch, Schmitz, and Seufert et al. (2017) on a 7-point Likert scale^44^.

A cross-sectional study (Study 1) followed 3 days later by a double-blinded randomised control trial (Study 2) with pre- and post-testing was designed to compare student’s learning performance and ICL/ECL. Experimental design and flow of activities of studies one and two is detailed in Fig. 1.

### Ethical approval

Ethical approval was obtained from the Nanyang Technological University Institute Review Board (IRB-2022-476). Study methods were performed in accordance with approved guidelines and regulations. Participation in the study was voluntary and would neither replace the existing cardiac and liver anatomy curriculum nor impact on formal assessment for the participants. Signed informed consent was obtained from participants prior to enrolment into the study. Confidentiality was ensured by de-identification of participants’ names during data collection. Singapore Dollar $10 vouchers were provided as tokens of appreciation.

### Participant recruitment

Participants were recruited from the first-year undergraduate medical student cohort of the Lee Kong Chian School of Medicine in Singapore. Fifty participants were recruited based on a similar power calculation to that used in the study by Skulmowski and Rey^13^.The study preceded formal teaching on cardiac and liver anatomy. The study was only publicised in the week leading up to the study through class announcement and email. Study 1 was conducted on 15 August 2022 and Study 2 was conducted on 18 August 2022.

### Mental rotations test

Participants completed the redrawn Vandenberg & Kuse (1978) mental rotations test (MRT), following protocols outlined by Peters et al.^43^. Responses were scored out of 48. Participants were sorted into equal groups of high or low SA by a median split depending on their relative scores upon completion of the MRT^45^.

### Pre-tests

Pre-tests (Appendix 1.1) were designed to assess participants’ baseline knowledge of cardiac (Study 1) and liver (Study 2) anatomy, utilising a closed-book format. Face validity of the question items were ensured by expert discussion, where questions were assessed to be appropriate to address the research aims.

### Learning phases and post-tests

**Figure 2:**
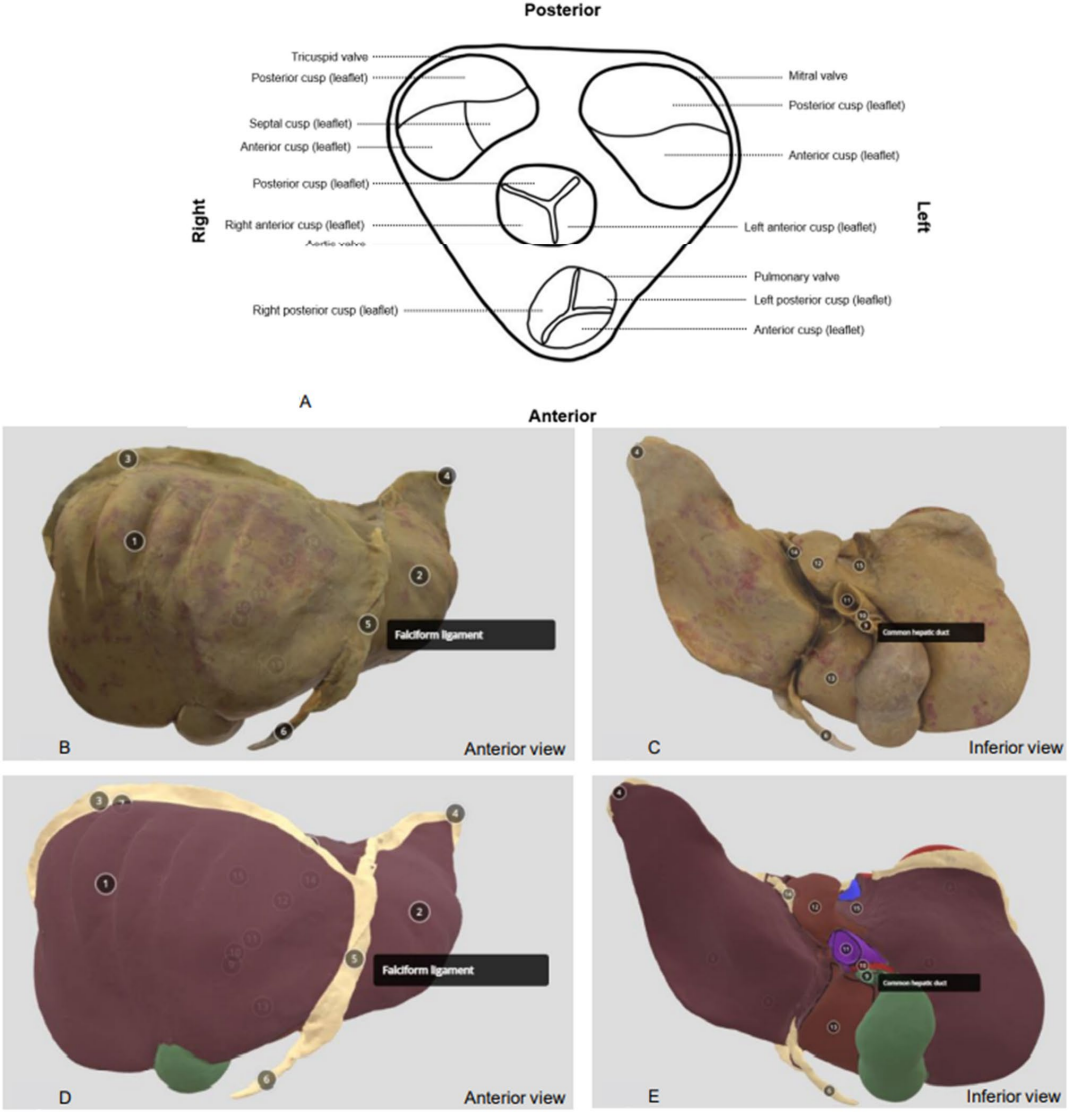
Learning materials provided to the participants. (**A**) 2D-labelled diagram depicting superior view of heart valves and their cusps. (**B**) Anterior view of Photorealistic 3D model of the liver. (**C**) Inferior view of Photorealistic 3D model of the liver. (**D**) Anterior view of Artistic 3D model of the liver with homogenised textures, colour-coding and outlining of structures in close proximity. (**E**) Inferior view of Artistic 3D model of the liver with homogenised textures, colour-coding and outlining of structures in close proximity.

Study 1: Participants had three minutes to memorise a 2D-labelled diagram of the superior view of heart valves (Fig. 2A), and were told to memorise the names, shapes, positions and orientation of the heart valves and cusps. Participants then had 10 min to sketch the same diagram and label the valves and their components in the opposite orientation to the diagram in the learning phase. This involved the participants mentally rotating the diagram 180 $^\circ$ to draw the valves. The paper provided to complete the drawing was fixed to the desk surface, such that participants were not able to rotate the paper nor change their seats while drawing. The drawings were collected, marked, and analysed according to a standardised scheme. Participants also answered additional questions on the difficulty of orienting and sketching the heart valves (Appendix 1.2).

Study 2: Participants were randomly assigned to one of two groups via block randomisation. One group was shown a photorealistic 3D model of the liver, while the other group was shown the artistic version of the photogrammetric model (A3DM). The resources were developed in-house via photogrammetry of plastinated liver specimen. The photographs of plastinated liver specimens (Von Hagens Plastination, Gubener Plastinate GmbH, Guben, Germany) were taken at every 5 degrees of rotation using an automated turntable. This was performed 4–5 times at different angles to capture all the specimen's details. The best 100 photos (the limit under Autodesk's educational license) were stitched together using Autodesk Recap Photo (Autodesk Inc., 2015, California, USA) to generate a 3D model. In Autodesk Maya (Autodesk Inc., San Rafael, CA), the 3D model was refined, and a texture map was applied to generate PR3DM. In A3DM, textures were homogenised and structures colour-coded using Blender (V3.2.1, Stichting Blender Foundation, Amsterdam)^46^ according to anatomy conventions^47^. The geometry, anatomical details, and labelling were constant between both models (Fig. 2B–E). An example of such labelling is indicated in Fig. 2B,D, for “Falciform ligament”, and in Fig. 2C,E, for “Common hepatic duct”. Lines were also drawn to differentiate the borders of different structures in the A3DM. Models were uploaded onto Sketchfab (V2.22.0, 2022 Sketchfab Inc, New York) for 3D visualisation and user-interaction.

Participants were instructed to memorise the names and positions of 15 labelled components of the liver, and their relationships to each other. In the study by Skulmowski, participants were given 90 s to study a 2D knee model with 16 labels^13^. Since the model in this study required rotation and touch to reveal labels, three minutes (180 s) were given to study the model. Participants then answered a 10-question (open-ended, different from pre-test) post-test, with diagrams from their respective models (Appendix 1.3). These questions assess the participants ability to identify the liver structures.

Face validity of both the drawing assessment for Study 1 and liver post-test questions for Study 2 were ensured by expert discussion, where questions were assessed to be appropriate to address the research aims. Questions of study 2 were also designed similarly to the school’s practical anatomy spot tests, of which the researchers have much experience in writing.

### Cognitive load questions

Following study 1’s learning phase, participants answered ICL questions from the survey instrument by Klepsch et al. (2017) on a 7-point Likert scale, and ECL questions for study 2^44^ (Appendix 1.4). This cognitive load instrument with excellent internal consistency is appropriate for learning using simple visualisations^48^. However, “task” was replaced with “visualisation” for more clarity^13^.

### Statistical analysis

Means and standard deviations were reported for normally distributed continuous variables, median and interquartile range for non-normal variables, and frequencies and percentages for categorical variables. Student’s t-test was used to compare normally distributed continuous variables, Mann–Whitney U test for non-normal variables, and chi-squared/Fisher’s exact test for categorical variables as appropriate. Pearson correlation was conducted for the relationship between MRT scores and drawing assessment scores. Cronbach $\alpha$ was used to determine survey internal consistency. Cohen’s D and $r = \frac{\left| z \right|}{{\sqrt n }}$ was used to determine effect sizes (ES). Data processing was performed through IBM SPSS Statistics Version 28.0 (IBM Corp. Released 2021. IBM SPSS Statistics for Windows, Version 28.0. Armonk), where *p* < 0.05 was statistically significant.

## Results

### Participant demographics

Fifty participants were recruited, and demographics are detailed in Table 1. No participant reported prior anatomy experience. The participants were grouped into low (MRT score $\le$ 26) and high (MRT score $>$ 26) SA based on the median score. 46 participants continued voluntarily with study 2, with 24 (50.0%) shown the PR3DM, and 22 (45.8%) shown the artistic version. There was no significant difference in cardiac and liver pre-test scores between groups divided based on SA and 3D model. The ICL/ECL measures had relatively good internal consistency (Cronbach $\alpha$: ICL 0.798, ECL 0.854).

**Table 1:** Demographics of the study cohort (n = 50).

Age, mean (S.D.)	19.4 (0.936)
Males, *n* (%)	31 (62.0)
Females, *n* (%)	19 (38.0)
Participants with no anatomy learning and training experience, *n* (%)	50 (100.0)
Mental rotations test score, median (IQR)	26.5 (20.75–38.25)
Participants shown photorealistic model, *n* (%)	24 (48.0)
Participants shown artistic model, *n* (%)	22 (44.0)

*IQR* Interquartile range, *S.D*. Standard deviation.

### Post-test performance and cognitive load

#### Study 1

The results from Study 1 are detailed in Table 2. There was no significant difference in overall ICL (*p* = 0.110) and difficulty of heart drawing (*p* = 0.187) between low and high SA groups. However, low SA participants reported significantly higher difficulty in remembering names of valves and cusps (*p* = 0.038). The low SA group scored significantly lower for overall heart drawing (*p* =  < 0.001, ES = 0.752), and for individual valves. MRT scores were moderately correlated with heart drawing scores (*r* = 0.407, *p* = 0.001).

**Table 2:** Comparisons between low and high spatial ability.

	Low SA (n = 25)	High SA (n = 25)	*p* value
Cardiac Pre-test Scores %, *mean (S.D.)*	21.7 $\pm$ 14.4	24.0 $\pm$ 18.8	0.631^a^ (ES: 0.137^c^)
ICL average*, mean (S.D.)*	4.34 $\pm$ 1.38	3.70 $\pm$ 1.40	0.110^a^
Additional questions on drawing difficulty, average, median (IQR)	2.75 (2.25–3.25)	2.5 (2.0–2.875)	0.187^b^
AddQ1, median (IQR)	3 (2–3.5)	3 (2–3)	0.695^b^
AddQ2, median (IQR)	3 (2–3.5)	2 (2–3)	0.038^b^
AddQ3, median (IQR)	3 (2–3.5)	2 (2–3)	0.194^b^
AddQ4, median (IQR)	3 (2–3.5)	3 (2–3)	0.644^b^
Heart valves drawing scores, median (IQR)	16.5 (7.5–24.0)	26.0 (21.0–30.0)	< 0.001^b^ (ES: 0.752^d^)
Pulmonary, median (IQR)	2.0 (2.0–6.0)	8.0 (6.0–8.0)	< 0.001^b^ (ES: 0.753^d^)
Aortic, median (IQR)	5.0 (2.0–6.0)	8.0 (6.0–8.0)	< 0.001^b^ (ES: 0.752^d^)
Tricuspid, median (IQR)	6.0 (0.0–8.0)	8.0 (6.0–8.0)	0.015^b^ (ES: 0.747^d^)
Mitral, median (IQR)	4.0 (0.0–6.0)	6.0 (4.5–6.0)	0.015^b^ (ES: 0.747^d^)

*AddQ1* Question on difficulty of drawing valves in reverse orientation, *AddQ2* Question on difficulty of remembering names of valves and cusps, *AddQ3* Question on difficulty of remembering position of cusps within valves, *AddQ4* Question on difficulty understanding relationship between valves, *ES* Effect size, *ICL* Intrinsic cognitive load, *IQR* Interquartile range, *S.D*. Standard deviation.

^a^Independent sample student’s t-test.

^b^Mann-Whitney U test.

^c^Cohen’s D for effect size (Effect size of > 0.25 considered moderate, > 0.5 considered large^49^).

^d^ $r = \frac{\left| z \right|}{{\sqrt n }}$ for effect size (Effect size of > 0.25 considered moderate, > 0.5 considered large^49^).

#### Study 2

The results from Study 2 are detailed in Table 3. There was no significant difference in ECL (*p* = 0.720) between PR3DM and A3DM groups. The A3DM group had significantly higher liver post-test scores than the PR3DM group (*p* = 0.042, ES = 0.523).

**Table 3:** Comparisons between low and high spatial ability; PR3DM and A3DM.

	PR3DM (n = 24)	A3DM (n = 22)	*p* value
Liver pre-test scores %, *mean (S.D.)*	6.77 ± 2.49	6.25 ± 2.70	0.532^a^ (ES: 0.042^b^)
ECL average*, mean (S.D.)*	3.44 ± 1.56	3.59 ± 1.19	0.720^a^
Liver post-test scores %, *mean (S.D.)*	30.83 ± 17.17	40.46 ± 19.63	0.042^a^ (ES: 0.523^b^)

*A3DM* Artistic 3D model, *ES* Effect size, *ECL* Extraneous cognitive load, *PR3DM* Photorealistic 3D Model, *S.D*. Standard deviation.

^a^Independent sample student’s t-test.

^b^Cohen’s D for effect size (Effect size of > 0.25 considered moderate, > 0.5 considered large^49^).

### Mental rotation test and sex differences

A stratified random sample of 19 males was obtained for comparison between sexes, to account for the large difference in numbers of female and male participants. There were significantly more males in the high SA group in both the original population and stratified sample, detailed in Table 4.

**Table 4:** Comparison between females and males, pre- and post-stratification.

Pre-stratification	Female (n = 19)	Male (n = 31)	*p* value
Low spatial ability, *n* (%)	13 (68.4)	12 (38.7)	0.041^a^
High spatial ability, *n* (%)	6 (31.6)	19 (61.3)	

Post-stratification	Female (n = 19)	Male (n = 19)	*p* value
Low spatial ability, *n* (%)	13 (68.4)	6 (31.6)	0.023^a^
High spatial ability, *n* (%)	6 (31.6)	13 (68.4)	

^a^Chi-squared test.

Details of comparison between females and stratified sample of males are indicated in Table 5. There were significantly more males in the high SA group (*p* = 0.023). Males also had significantly higher MRT scores than females (*p* = 0.011, ES = 0.779). Otherwise, there were no significant differences in heart drawing scores (*p* = 0.254) and ICL (*p* = 0.065) for Study 1, and ECL (*p* = 0.099) and liver post-test scores (*p* = 0.263) for Study 2 between sexes.

**Table 5:** Comparison between females and stratified sample of males.

	Female (n = 19)	Male (n = 19)	*p* value
MRT Score,* mean (S.D.)*	23.7 $\pm$ 10.8	31.7 $\pm$ 9.95	0.011^a^ (ES: 0.770^d^)
Low spatial ability, *n* (%)	13 (68.4)	6 (31.6)	0.023^c^
High spatial ability, *n* (%)	6 (31.6)	13 (68.4)	
ICL average, *units, mean (S.D.)*	4.61 $\pm$ 1.49	3.92 $\pm$ 1.23	0.065^a^
Heart drawing scores, median (IQR)	19.5 (14.0–26.0)	25.0 (18.0–30.0)	0.254^b^ (ES: 0.035^e^)
Pulmonary, median (IQR)	2.0 (2.0–7.0)	7.5 (5.5–8.0)	0.050^b^ (ES: 0.675^e^)
Aortic, median (IQR)	6.0 (2.0–8.0)	8.0 (3.75–8.0)	0.267^b^ (ES: 0.033^e^)
Tricuspid, median (IQR)	6.5 (5.0–8.0)	5.0 (5.0–8.0)	0.779^b^ (ES: 0.002^e^)
Mitral, median (IQR)	6.0 (2.0–6.0)	6.0 (2.0–6.0)	0.818^b^ (ES: 0.002^e^)
Additional questions on drawing difficulty, average, median (IQR)	2.75 (2.25–3.25)	2.75 (2.0–3.0)	0.841^b^
AddQ1, median (IQR)	3 (2–3)	3 (2–3)	0.998^b^
AddQ2, median (IQR)	3 (2–3)	2 (2–3)	0.596^b^
AddQ3, median (IQR)	2 (2–3)	3 (2–3)	0.728^b^
AddQ4, median (IQR)	3 (2–3)	3 (2–3)	0.522^b^
ECL average*, mean (S.D.)*	3.09 $\pm$ 1.59	3.70 $\pm$ 1.23	0.099^a^
Liver post-test scores %, *mean (S.D.)*	35.6 $\pm$ 22.5	31.5 $\pm$ 14.6	0.263^a^ (ES: 0.216^d^)

*AddQ1* Question on difficulty of drawing valves in reverse orientation, *AddQ2* Question on difficulty of remembering names of valves and cusps, *AddQ3* Question on difficulty of remembering position of cusps within valves, *AddQ4* Question on difficulty understanding relationship between valves, *ES* Effect size, *ECL* Extraneous cognitive load, *ICL* Intrinsic cognitive load, *IQR* Interquartile range, *MRT* Mental rotations test, *S.D*. Standard deviation.

^a^Independent sample student’s t-test.

^b^Mann-Whitney U test.

^c^Chi-squared test.

^d^Cohen’s D for effect size (Effect size of > 0.25 considered moderate, > 0.5 considered large^49^).

^e^ $r = \frac{\left| z \right|}{{\sqrt n }}$ for effect size (Effect size of > 0.25 considered moderate, > 0.5 considered large^49^).

## Discussion

Through this study, it was found that (1) participants with low SA had significantly lower heart drawing scores than those with high SA despite no significant differences in reported ICL, (2) males had significantly higher SA than females, (3) there were no significant differences in reported ECL when signalling in the form of colour-coding was added to the PR3DM, and (4) participants who studied the liver A3DM had significantly higher post-test scores than those who studied the liver PR3DM. The findings are important and provide useful insights on the impact that spatial ability and photorealistic and artistic 3D models have on anatomical education and their application to the research of instructional and assessment design in anatomy.

The finding that participants with a low SA had significantly lower heart drawing scores supported our first hypothesis that learners with a high SA have superior anatomy performance in drawing assessment. The correlation between MRT and heart drawing scores was moderately positive. This corroborates with the meta-analysis by Roach et al. reporting significant positive pooled correlation between SA and anatomy performance when drawing tasks were used to assess anatomical knowledge^50^. From our study, it was found that males had significantly higher SA than females (*p* = 0.011), and there were significantly more males in the high SA group (*p* = 0.023). As modalities like drawing rely more on spatial reasoning, they can exacerbate effects favouring higher SA students, such as males. This emphasizes that anatomy assessment should be designed from both spatial and non-spatial perspectives, especially for anatomy beginners where SA has a greater effect on anatomy performance^51,52^. In several studies, SA improved with repetition and practice^53–55^, and mentored sketching (in engineering fields)^56^. For example, Provo et al. (2002) found that males had superior spatial ability, but that at 8 months, spatial abilities were comparable^42^. Our findings suggest that drawing can be encouraged during anatomy teaching and learning sessions to promote SA, increasing experiences of knowledge construction. However, this should be further investigated.

A possible explanation for the low SA group's performance in heart drawing is that they were unable to mentally imagine, orient, and map the heart valves with their components from an unfamiliar perspective. This impacted their ability to recall and present the image correctly. Previous research found that participants who could not draw objects from an imagined viewpoint could do it from their actual viewpoint^57^. This indicates that a deficiency in drawing abilities should not be a problem in recalling the image. Therefore, using drawing in this study as an assessment tool to evaluate spatial knowledge is reasonable. Interestingly, ICL was not significantly different between low and high SA. The low SA group’s self-reported scores on the difficulty of drawing in different orientation and remembering valve and cusp positions were comparable to the high SA group. This phenomenon could possibly be explained by the Dunning-Kruger effect, where students who perform poorly overestimate their performance in self-ratings^58^, which has been observed in the medical field^59,60^. However, low SA participants reported much increased difficulty remembering cardiac valve and their cusp names. Engaging in active learning techniques such as sketching and labelling of the cardiac valves and cusps can help students’ memory and retention of information.

Our second hypothesis was that colour-coded 3D model would decrease ECL. Previously, literature showed that PR3DMs can provide accurate details^10^ but can increase ECL due to their anatomical and textural details that may not be essential to learning^12,61^. Significant ECL reduction through colour coding has also been reported^14^. In this study, non-additive signalling—colour-coding—was added, highlighting structural differentiation^28^. However, the current study observed no significant difference in ECL when PR3DM was colour coded. The reason for this could be because the distinct colours for each structure in the liver A3DM coupled with outlines of structures that were in close proximity to each other reduced ambiguity in determining the borders of anatomical structures, mitigating the increased cognitive load from photorealistic geometric and structural details even though textural and colour realism had to be sacrificed. Another possible reason for the similar ECL is that other factors contributing to ECL were kept constant between groups in the current study. For example, the transient information effect, describing provision of free-control of dynamic visual aids, also interferes with learning spatially-complex information^62^. This effect was kept constant as participants studying both models could independently control the rotation, size, and revelation of labels. Another example would be the redundancy effect, elicited as both 3D models were viewable from all directions, where presenting extra materials like multiple spatially-challenging views increases ECL and decreases working memory^63^, affecting anatomy learning. It was also reported that non-essential information should be discarded from visualisations, with only key structural views presented, especially in initial learning phases^64,65^. Further studies can be conducted to investigate how presenting a series of static key views with sacrificed user-interactivity compares with freely rotatable PR3DMs with good interactivity but presents all views (including non-essential ones).

Perhaps what is most important is the impact of instructional design on learning performance, as per our third hypothesis. Our results demonstrated that participants who studied the A3DM performed significantly better in post-testing than the PR3DM group, suggesting that colour-coding translated to better recognition, distinction of structures and information retention. Photorealism is useful because it preserves true geometry, giving accurate representations of subject material^10^. Our findings substantiate that PR3DM enhancement through signalling may increase the learner’s attention and interest on the 3D anatomy models. This may have implications for the creation of learning tools and instructional design, in that realistic and color-coded 3D anatomy models can be displayed in tandem when guiding students in learning human anatomical regions that are more difficult and complicated to understand. However, our findings must be taken with the caveat that in the post-test, question diagrams corresponded to the model the participants studied. The question thus arises whether visual aids inadvertently increased the reliance of learners on these cues, potentially lowering their performance when tested without cues.

## Limitations

We acknowledge several study limitations. Firstly, Study 1 was limited to the heart and Study 2 was limited to the liver. Future larger studies should evaluate if similar effects can be replicated with other human anatomical regions and organs. Secondly, learning strategies between the high and low SA groups could not be delineated and may have been different. Given the short learning phase, low SA participants may simply lack time to generate effective visualisation strategies to retain knowledge. Eye motion analyses would be helpful in identifying differences in viewing patterns, providing clearer explanations on how the high SA group performed better. Thirdly, self-selection bias may be another limitation in that students interested to learn anatomy before the formal course may have participated compared to those uninterested. Lastly, this investigation was undertaken prior to the formal anatomy course, thus limiting its validity to untrained individuals. It is possible that experienced learners may develop mechanisms to compensate for decreased spatial ability, and further studies in this cohort may be useful.

## Conclusions

This investigation demonstrated the positive effects of SA and colour-coding on anatomy performance. The participants in the photorealistic and artistic 3D groups indicated similar ECL. These may have significance for the instructional and evaluation design based on PR3DMs and techniques of drawing anatomy.

## Supplementary Information

Supplementary Information.

## Data Availability

The datasets used and/or analysed during the current study available from the corresponding author on reasonable request.
