# Supplementary material for: Spatial ability and 3D model colour-coding affect anatomy performance: a cross-sectional and randomized trial
Source: Sci Rep. 2023 May 15;13:7879. doi: 10.1038/s41598-023-35046-2 (PMC10185657; doi:10.1038/s41598-023-35046-2)
Supplement: Supplementary file 1 — Supplementary Information. [file 41598_2023_35046_MOESM1_ESM.docx]

## Appendix 1 - Supplementary material

**Appendix 1.1 Pre-test Questions**

Cardiac Pre-test

*Marks in [square brackets]*

1. During systole, which valve prevents the backflow of blood from the right ventricle to the right atrium? **Answer: Tricuspid valve [1]**
2. Name all the valves of the heart that have 3 cusps (leaflets). **Answer: Aortic valve [1], Pulmonary valve [1], Tricuspid valve [1]**
3. What is the shape of the cusps (leaflets) of the aortic valve? **Answer: Semi-lunar / half moon-shaped [1]**
4. *Fill in the blank with an appropriate word/phrase describing the relative position of the valves to each other.* The aortic valve is situated ______ the pulmonary valve. **Answer: Posterior to / behind [1]**
5. *Fill in the blank with an appropriate word/phrase describing the relative position of the valves to each other.* The mitral valve is situated ______ the tricuspid valve. **Answer: On the left of / left to [1]**
6. Identify the indicated vessel. (Image with arrow pointing to right coronary artery provided.) Answer: **Right coronary artery [1]**
7. What structure is immediately to the left of vessel “X”? (Image with left common carotid artery indicated as “X” provided.) Answer: **Left subclavian artery [1]**
8. Vessel “X” arises from which structure? (Image with pulmonary trunk indicated as “X” provided.) **Answer: Right ventricle [1]**
9. Identify structures Y and Z respectively. (Image with left atrium indicated as “Y” and left ventricle indicated as “Z” provided.) **Answer: Y - Left atrium [1]; Z – Left ventricle [1]**
10. Name the vessel indicated by the black arrow. (Image with arrow pointing to left anterior descending artery provided.) **Answer: Left anterior descending artery / anterior interventricular artery [1]**

Liver Pre-test

*Marks in [square brackets]*

1. Name the 4 anatomical lobes of the liver. **Answer: Left [1], Right [1], Quadrate [1], Caudate [1] lobes**
2. Name any one peritoneal ligament of the liver. **Answer: Falciform / Coronary / Triangular ligament [1]**
3. Name any embryological ligament (remnant) associated with the liver. **Answer: Ligamentum teres / Ligamentum venosum [1]**
4. Through which area do structures enter and leave the liver? **Answer: Porta hepatis [1]**
5. What vessel contributes about 70% of blood supply to the liver? **Answer: Portal vein [1]**

**Appendix 1.2 Additional questions on difficulty of heart drawing**

1. How easy/difficult was it to draw the valves of the heart in a reverse orientation from the learning phase?
2. How easy/difficult was it to remember the names of the different valves and their cusps/leaflets?
3. How easy/difficult was it to remember the position of the different cusps/leaflets within each heart valve?
4. How easy/difficult was it to understand the relationship between the different heart valves?

Rated on a 5-point scale:

1 – Very easy, 2 – Easy, 3 – Somewhat difficult, 4 – Very difficult, 5 – Unable to do this

**Appendix 1.3 Liver Post-test Questions**

Liver Post-test

*Marks in [square brackets]*

1.
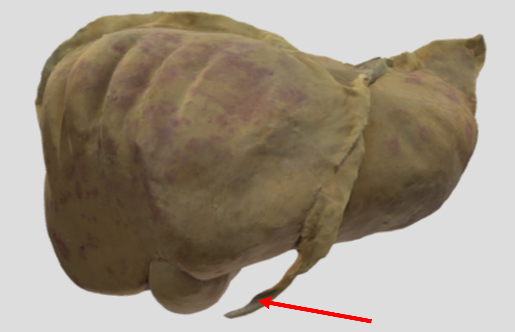

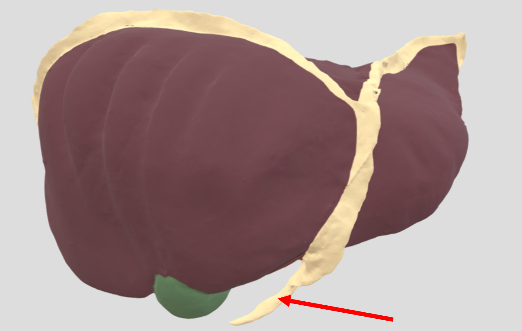


Identify the indicated structure. **Answer: Ligamentum teres [1]**

1.
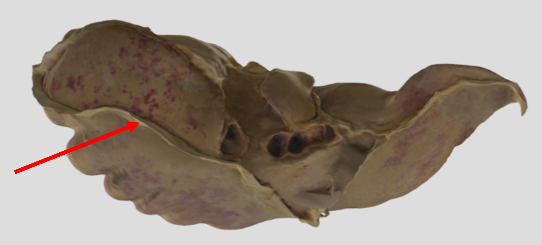

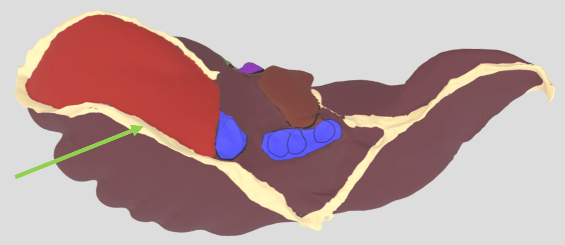


Identify the indicated structure. **Answer: Coronary ligament [1]**

1.
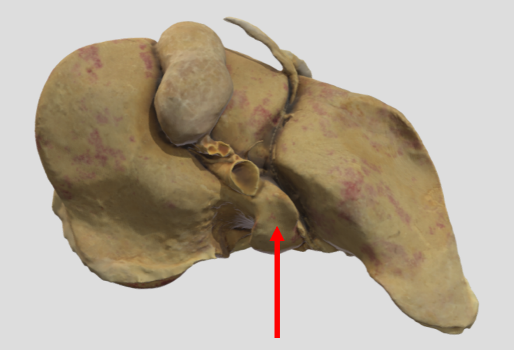

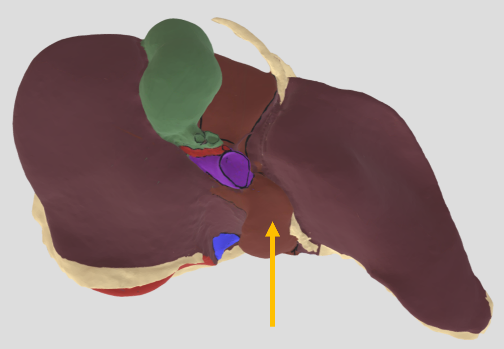


What structure is immediately to the right of the indicated (arrow)? **Answer: Groove for inferior vena cava [1]**

1.
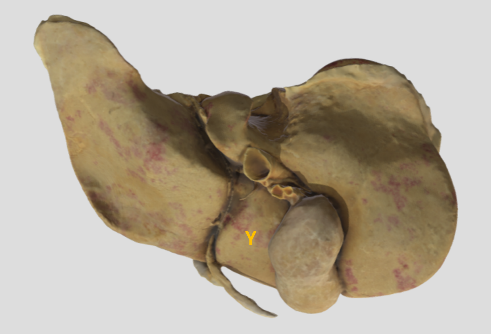

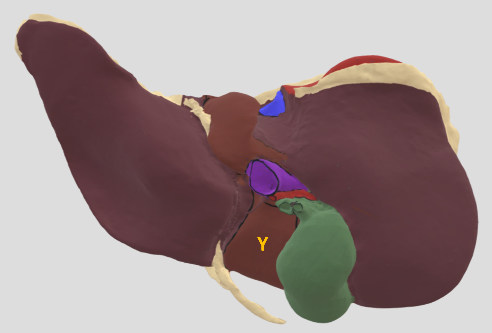


Name structure Y. Answer: **Quadrate lobe [1]**

1.
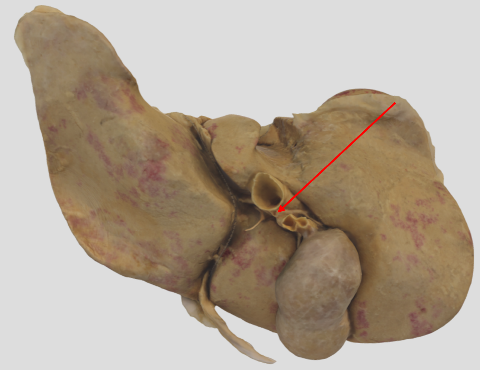

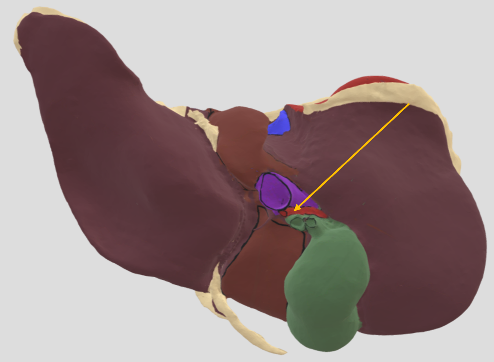


What is immediately posterior to the indicated structure? **Answer: Portal vein [1]**

1.
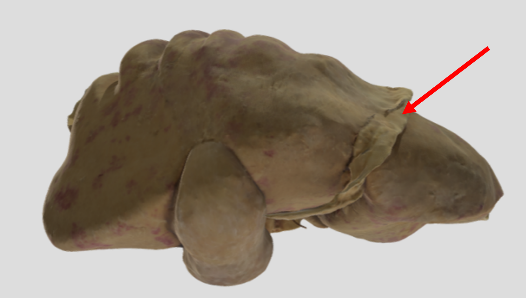

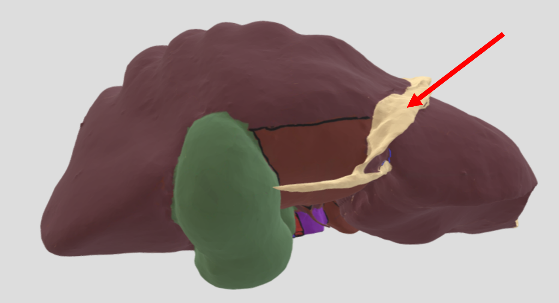


Identify the indicated structure. **Answer: Falciform ligament [1]**

1.
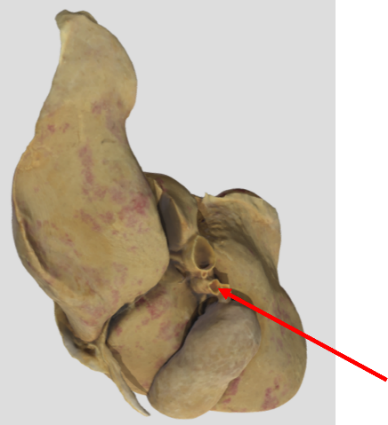

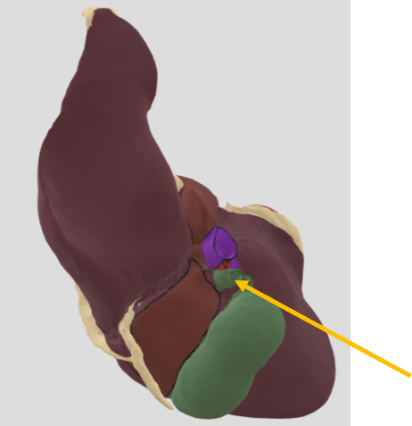


Name the indicated structure. **Answer: Common hepatic artery [1]**

1.
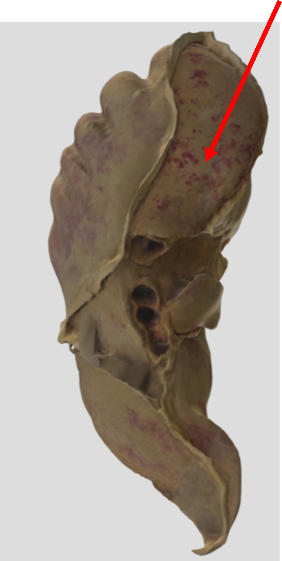

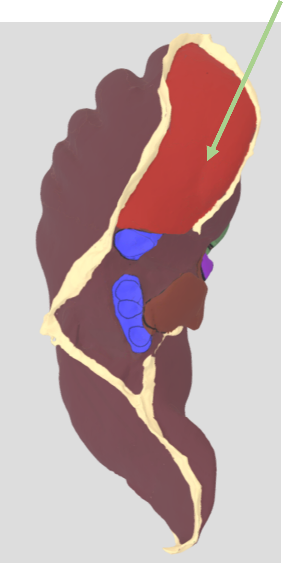


Name the indicated part of the liver. **Answer: Bare area [1]**

1.
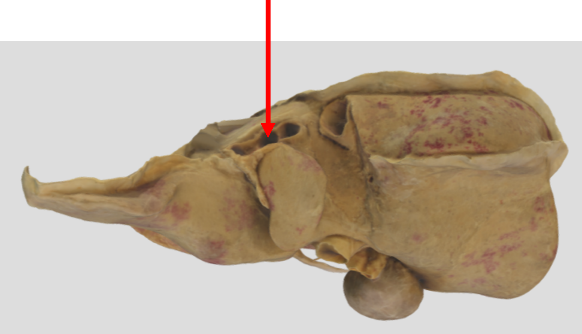

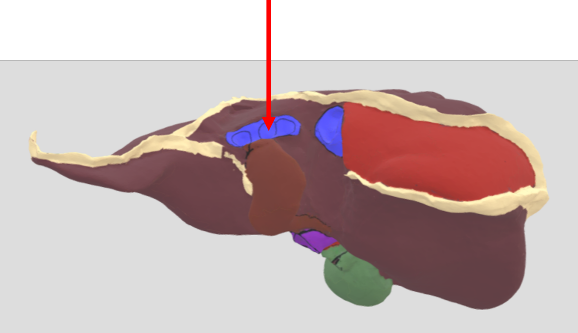


Identify the indicated structure. **Answer: Hepatic veins [1]**

1.
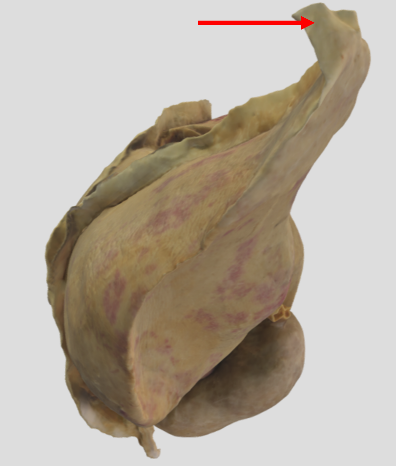

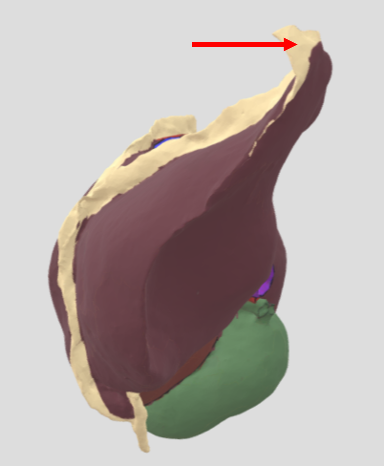


Identify the indicated structure. **Answer: Left triangular ligament [1]**

**Appendix 1.4 Cognitive load survey items**

ICL:

1. For this visualisation, many things needed to be kept in mind simultaneously.
2. This visualisation was very complex

ECL:

1. During this visualisation, it was exhausting to find the important information.
2. The design of this visualisation was very inconvenient for learning.
3. During this visualisation, it was difficult to recognise and link the crucial information.

Rated on a 7-point Likert scale:

1 – Strongly disagree, 2 – Disagree, 3 – Somewhat disagree, 4 – Neither agree nor disagree, 5 – Somewhat agree, 6 – Agree, 7 – Strongly agree
